# Continuous glucose monitoring in preterm infants: evaluation by a modified Clarke error grid

**DOI:** 10.1186/s13052-016-0236-9

**Published:** 2016-03-09

**Authors:** Eloisa Tiberi, Francesco Cota, Giovanni Barone, Alessandro Perri, Valerio Romano, Rossella Iannotta, Costantino Romagnoli, Enrico Zecca

**Affiliations:** Division of Neonatology, Department of Pediatrics, UniversityHospital “A.Gemelli” CatholicUniversity of the Sacred Heart, Rome, Italy

**Keywords:** Continuous glucose monitoring, Dysglycaemia, Preterm infants

## Abstract

**Background:**

Continuous glucose monitoring using subcutaneous sensors has been validated in adults and children with diabetes, and was found to be useful in the management of glucose control. We aimed to assess feasibility and reliability of a new continuous glucose monitoring system (CGMS) in a population of preterm neonates using a Clarke error grid (CEG) specifically modified for preterm infants.

**Methods:**

Preterm infants were recruited within 24 h from delivery. A subcutaneous sensor connected to a CGMS was inserted and maintained for 6 days. Data collected from CGMS were compared with data obtained using a glucometer. Management of the infants followed standard protocols and was not influenced by CGMS readings.

**Results:**

Twenty patients (9 males) were included. Median (range) gestational age was 32 weeks (27–36) and median (range) birth weight was 1350 g (860–3360). Average CGMS recording time was 137 h, for a total of 449 paired glucose levels. CEG and modified CEG criteria for clinical significance were met.

**Conclusion:**

CGMS is a safe and clinically adequate method to estimate glucose levels in preterm infants. As the glucose level can be evaluated in real time, this CGMS could be useful to reduce the number of heel sticks, to observe glycaemic trends and to promptly detect episodes of both hypo- and hyper-glycaemia.

## Background

Preterm newborns are particularly exposed to blood glucose levels fluctuations after birth, and their developing brain is thought to be more susceptible to episodes of both hypo- and hyper-glycaemia [1]. Such episodes, although asymptomatic, usually occur during the first week of life and are often clinically relevant. Symptomatic hypoglycaemia has been associated with poor neurodevelopmental outcome [1, 2]. Despite the relevance of these metabolic alterations, their assessment is still hindered by the lack of appropriate tools to monitor glucose levels continuously [3, 4]. Plasma glucose levels, obtained through enzymatic methods and performed in central laboratory facilities, remain the gold standard for glucose assessment in the newborn [5]. This requires either repeated blood draws or manipulation of central lines, which should be avoided in preterm infants in order to reduce risk of infections and anemia. Newborn infants at high risk for dysglycaemia are usually monitored several times per day, through finger-stick capillary blood and point-of-care (POC) blood glucometer (GTX) [6]. Checking blood glucose levels intermittently, however, could lead to undetected episodes of altered glycaemia. For this reason, continuous glucose monitoring systems (CGMSs) using subcutaneous sensors have been validated in adults and children with diabetes, and have been shown particularly useful in the management of glucose control [7]. Three different devices have been approved by the US Food and Drug Administration for continuous glucose monitoring in newborns but none of them provides blood glucose values in real time on the monitor [8, 9]. Medtronic has recently produced a new generation instrument whose characteristics seem appropriate for neonatal intensive care unit. The present study was designed to assess feasibility and reliability of this new CGMS in a population of preterm infants.

## Methods

This non-randomized feasibility study was conducted between April 1, 2012 and September 30, 2012 in the neonatal intensive and sub-intensive care facilities of Catholic University A. Gemelli Hospital following the approval of our institutional review board. The following preterm infants at increased risk for neonatal dyslycemia were eligible for this study: intrauterine growth-restricted (IUGR) infants, small for gestational age infants (SGA), very low birth weight (VLBW) infants, extremely low birth weight (ELBW) infants, infants of diabetic mothers, maternal treatment with beta-blockers, tocolytics, oral hypoglycemic therapy, large for gestational age (LGA) infants, asphyxiated infants, septic infants, polycythaemic infants, infants with feeding difficulties.

Infants with major congenital abnormalities at birth or with skin diseases were excluded. Informed consent was obtained from the parents, and infants enrolled were connected to the CGMS (Medtronic, Northridge, Calif., USA) within the first 24 h of life. Each bedside blood glucose test was prescribed by the treating physician in accordance with the standards of clinical practice currently followed inour department. Management of the infants was not influenced by the CGMS readings.

The CGMS is composed by the ENLITE sensor, the Mini Link transmitter and the VEO monitor.

The ENLITE sensor has a cannula length of 8.75 mm and it is a new generation sensor. No other studies about CGMS reported the use of this device. It is equipped with a glucose-oxidase which, in the presence of glucose in the interstitial space, generates an electrical current every 10 s and transmits wirelessly it through a Mini Link transmitter to the VEO monitor that calculates the average of the currents measured every 5 min. The measuring principle is based on the generation, by the enzymatic activity of glucose-oxidase, of hydrogen peroxide from glucose and oxygen. The hydrogen peroxide is then oxidized by a specific electrode that triggers the movement of electrons. The measured current is then converted into an estimate of blood glucose through the calibration procedure. The system requires to be calibrated at least every 12 h, using the glucometer blood glucose values. The interstitial glucose concentration values are expressed in mg/dl in a range between 40 mg/dl (2.2 mmol/l) and 430 mg/dl (24 mmol/l), and values that do not fall within this range are expressed respectively as <40 mg/dl or >430 mg/dl while the VEO monitor gives a sound alarm. Data are monitored and showed in real time. Insertion procedure involves identification of an unharmed skin area in the lateral part of the thigh and application of a 2.5 % Lidocaine – 2.5 % Prilocaine ointment over that area 30 min before insertion of thesensor ENLITE. The sensor is introduced following a sterile procedure and then connectedto the MINILINK transmitter.

The personal and clinical data of each patient were collected in a dedicated database. Values recorded through the continuous monitoring software were downloaded via Care Link TM after sensor removal. Hypoglycaemia was defined as a glucose value ≤45 mg/dl [10–12], and hyperglycaemia as a glucose value ≥180 mg/dl [13]. Data obtained with the CGMS were compared with those obtained by GTX. We preferred a point of care method, GTX, as a comparison, to a more accurate laboratory method, because the first is the most used in clinical practice in the NICU.

### Statistics

As this was a feasibility study no formal sample size calculation could be applied. To visualize the agreement between the two measuring techniques we used the Bland-Altman plot, and the Clarke Error Grid (CEG) [14]. The CEG was specifically modified in order to be clinically relevant in the management of dysglycaemia in preterm infants.

Definitions of neonatal hypoglycemia and hyperglycemia requiring a therapeutic treatment were used to obtain modified CEG.

CEG evaluates the accuracy of a new tool, as compared to a standard method, for the determination of glycaemia in a clinical setting. It shows the value generated by the monitoring system being tested along the ordinate axis, and the measurement of glucose as obtained with the reference technique along the abscissa axis. The CEG identifies 5 areas with different error in accuracy combined with the severity of clinical consequences.

- **Region A**: values within 20 % of the reference sensor.
- **Region B**: values outside 20 % of the reference sensor, but that would not lead to an inappropriate treatment.
- **Region C**: values leading to an unnecessary treatment.
- **Region D**: values indicating a potentially dangerous failure to detect hypo- or hyper-glycaemia.
- **Region E**: values that would confuse treatment of hypoglycemia for hyperglycemia, and vice-versa.

Reference blood glucose levels in the standard CEG are those generally used in adult or pediatric patients. For this reason we chose to modify the grid according to the blood glucose levels used in the clinical management of newborn infants. In this modified Clarke error grid (MCEG) we set the limit for the diagnosis of hypoglycemia at 45 mg/dl, and the limit for the diagnosis of hyperglycemia to 180 mg/dl, because these values require therapeutic measures in the neonates, regardless of gestational age of the baby. As a consequence, the geometry of the region C upper size appears different from the original CEG.

## Results

The study population consisted of 20 infants, 9 males and 11 females, with a median gestational age of 32 weeks (range 27–36 weeks) and median birth weight of 1350 g (range 860 to 3360 g). The sensor was held in place with an average duration of 137 h. Nine patients were monitored performing 2 calibration procedures per day (every12 h), and 11 patients by performing 3 calibrations per day (every 8 h). An average number of 1637 measurements of blood glucose concentration from the interstitial spacewere obtained for each patient. On all 20 patients it was possible to obtain 449 pairs of VEO vs. GTX measurements.

**Fig. 1:**
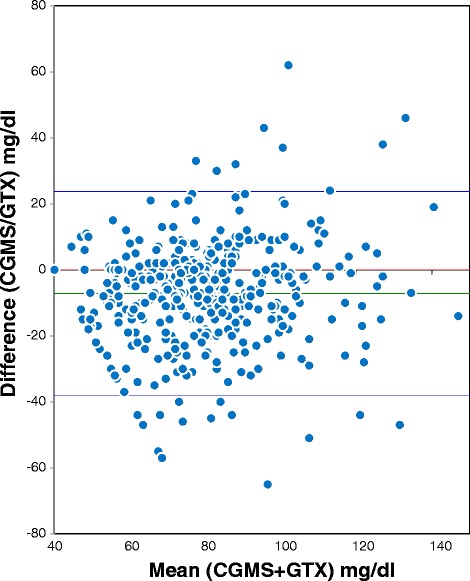
Bland Altman plot. CGMS vs GTX

Figure 1 reports the Bland Altman Analyses for all glucose measurements. The mean (95 % CI) difference was −6,8 (−37,4 to 23,8) mg/dl. The instrument shows a slight tendency to underestimate the value of blood glucose.

**Fig. 2:**
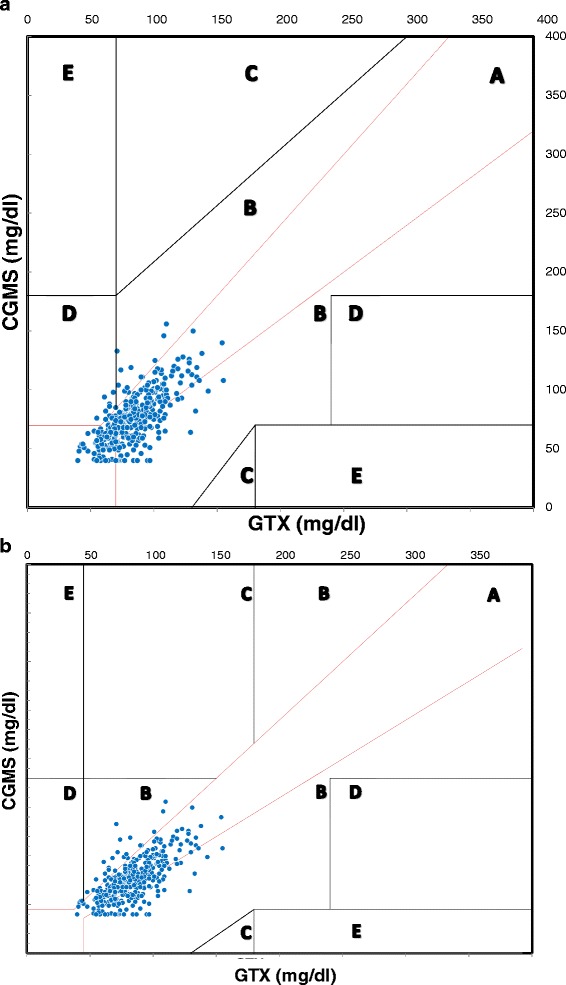
Clarke Error Grid CGMS vs GTX (**a**) MCEG CGMS vs GTX (**b**)

**Fig. 3:**
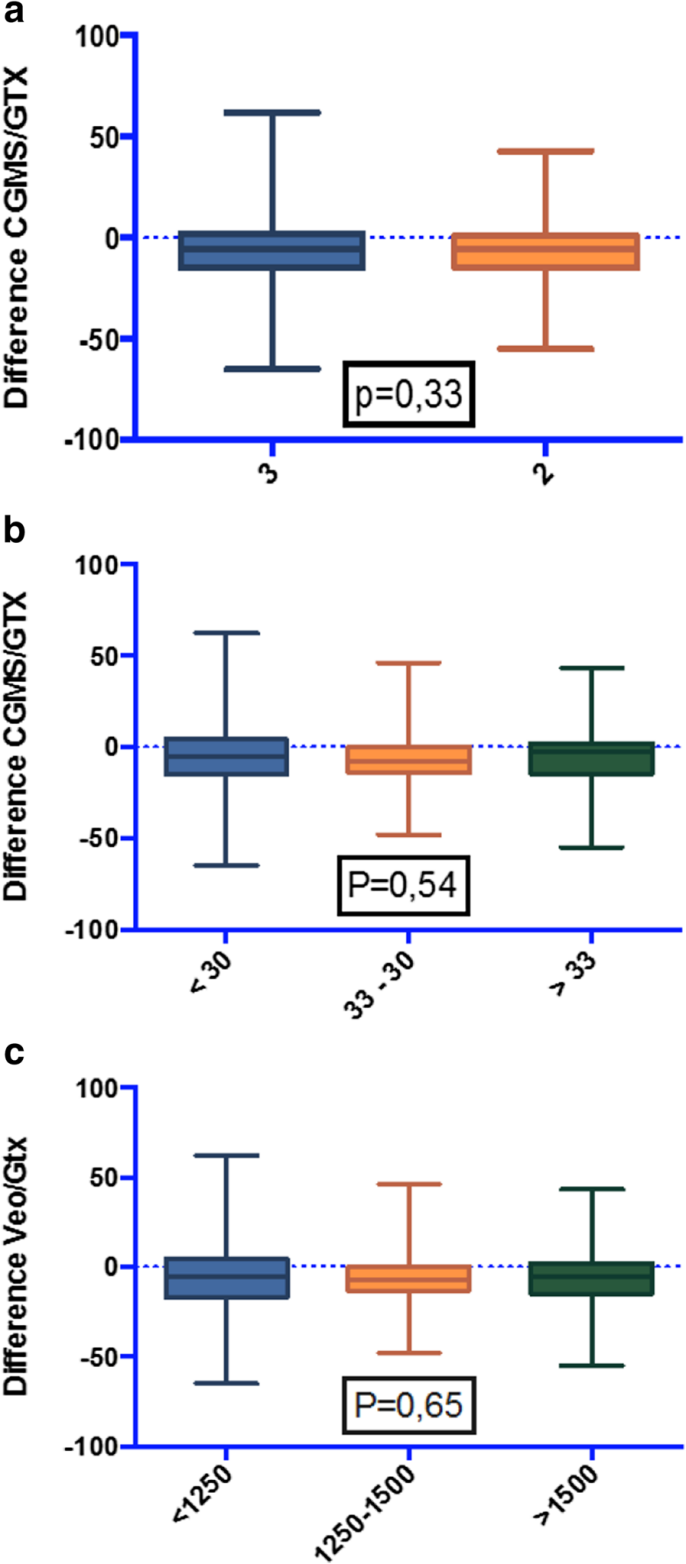
Subgroups analysis. **a** 2 vs 3 daily calibrations. **b** Gestational age soubgroups. **c** Birth weight soubgroups. Central bars indicate the average error. Range, upper and lower limits, and P values are also shown

Figure 2 reports the standard CEG and the MCEG. In the CEG, 78,2 % of measurements falls in region A, 20,0 % of measurements falls in region B, and 1,78 % of measurements in region D, without any value in region C or E. Differently, the MCEG shows 72,6 % of measurements in region A, 26,7 % of measurements in region B, 0,67 % of measurements in Region D and, again, not a single value falls in either region C or E. In order to evaluate whether sensor accuracy was influenced by gestational age, birth weight and number of calibrations, we carried out a subgroups analysis. The average error for the subgroups and the p value (Student *t* test and ANOVA) were evaluated. No statistically significant differences between the subgroups were identified (Fig. 3). It is to be mentioned that glucose values obtained during the study did not fall under the lower limit tested by CGMS.

## Discussion

We have tested a new generation device (CGMS, MiniMed, Medtronic, Northridge, Calif., USA) to continuously monitor blood glucose in preterm infants at risk of dysglycaemia. Three CGMS have already been tested in clinical practice and have been approved by the US Food and Drug Administration in newborns: DexCom Seven (DexCom Inc.), Guardian RT (Medtronic Diabetes, Inc.), and Navigator 40 (Abbott Diabetes). None of them, although, provides blood glucose values in real time on the monitor and uses the Enlite sensor, which seems to be more suitable for preterm infants [8, 9].

Bearsdall et al. performed a CEG comparing glucose values obtained from a previous Medtronic device with values obtained from different POC blood glucose tests and found great correlation between the two methods [15]. However, the standard CEG was firstly used to test the accuracy of blood glucose measurement in adults or children with diabetes [14]. Therefore, as pointed out by the same authors, the interpretation of these results in the setting of neonates with different interventional glucose levels needs to be cautious [15]. To our knowledge, this is the first report of a comparison between blood glucose values obtained via CGMS and via GTX using a specifically modified CEG, more suitable for preterm infants in the first days of life. Our results confirm a good correlation between CGMS and POC GTX, based on the established criteria that >95 % of all readings must be in either zone A or B. There is no evidence, in fact, in the MCEG, of an increase of the measurements into zones (C, D, E), that would lead to inadequate management of hyper- or hypo-glycaemia.

These data suggest that the CGMS is a clinically accurate instrument to evaluate blood glucose levels in preterm infants.

Our Bland Altman analyses for all glucose levels demonstrated that the CGMS slightly under-reads as compared to POC GTX, as has been previously reported [16, 17].

The new ENLITE sensor transfers a wireless signal directly to the VEO monitor, providing real time evaluation of blood glucose values. Out data suggest that, once the sensor is in place, 2 POC determination of blood glucose per day appear to be sufficient to calibrate the CGMS. We believe that this new CGMS has a great potential in reducing the number of heel sticks the newborn has to undergo for routine glucose determination.

The application of the sensor appeared to be safe and well tolerated, infants appeared to be unconcerned by its continuous presence throughout the study period. It remained effective after up to six days after positioning and it did not interfere with nursing care. Although there could be concerns about the use of this sensor in babies with very delicate skin, no evidence of significant tissue damage after placement for up to six days has been noted.

Previous studies showed that CGMS data correlates well with point of care devices, with minimal bias. We believe however that the clinical advantage of the use of CGMS in preterm infants would not be based on providing accurate single values but on showing a continuous information on trends of glucose control. This could lead to select the cases actually deserving a blood sample, limiting them only when really necessary, thus optimizing glucose control in preterm infants.

An important limit of the instrument is to provide measurements of blood glucose only in the range between 40 mg/dl (2.2 mmol/l) and 430 mg/dl (24 mmol/l), thus reducing the possibility of its use for clinical management of neonatal hypoglycaemia.

In effect, the value of continuous glucose monitoring should not be the ability of it to diagnose hypoglycemia but to give a warning of alterations in trends of glucose control. The finding of a value <40 mg/dL should lead to obtain a blood sample to assess the severity of hypoglycemia.

A limitation of our study is the small size of the population. However, the observational design of the study permitted to specifically address the efficacy and safety of the CGMS. In fact, we chose to compare blood glucose data provided by VEO with values provided by a single bedside glucometer, as opposed to other studies that have used different POC devices and even different kind of samples (capillary vs arterial sticks) [15].

We choose to include in our study preterm infants needing tight glucose control because of the increased risk of both hyperglycaemia and hypoglycaemia. Although our results fall within the range of normal glucose values, not allowing to prove the therapeutic application of the instrument, they still enable us to verify the feasibility and reliability of this new CGMS in our population of preterm infants.

Anyway, since all our data were substantially within the range of normal blood glucose concentrations and since the CGMS we tested is not able to read glucose levels lower than 40 mg/dl, further improvement of the sensor is needed before it can become a fundamental tool in the management of hypoglycemia. Despite these limits, our study shows that the CGMS in preterm infants allows caregivers to: 1) evaluate glucose concentration (mg/dl or mmol/liter); 2) detect trends in glucose levels (increasing, decreasing, or stationary); and 3) evaluate rate of glucose modifications [18].

The clinical use of sensors for continuous glucose monitoring could therefore improve management of glucose control in neonatal intensive care units, avoiding undetected pathological glucose fluctuations [19].

## Conclusions

Continuous glucose monitoring using subcutaneous sensors has been validated in adults and children with diabetes, and was found to be useful in the management of glucose control.

It is safe and reliable in preterm infants. It is useful to reduce the number of heel sticks, to observe glycaemic trends and to promptly detect episodes of both hypo- and hyper-glycaemia.

Although the present data are promising, studies with a larger population of preterm infants at increased risk of both hyperglycemia and hypoglycaemia, evaluating also abnormal glucose values, are required in order to better establish safety, reliability and therapeutic applications of the CGMS.

## Abbreviations

CEG: clarke error grid
CGMS: continuous glucose monitoring system
